# Association of miR-21-5p, miR-122-5p, and miR-320a-3p with 90-Day Mortality in Cardiogenic Shock

**DOI:** 10.3390/ijms21217925

**Published:** 2020-10-26

**Authors:** Mikko Hänninen, Toni Jäntti, Heli Tolppanen, Heli Segersvärd, Tuukka Tarvasmäki, Johan Lassus, Mélanie Vausort, Yvan Devaux, Alessandro Sionis, Ilkka Tikkanen, Veli-Pekka Harjola, Päivi Lakkisto, for the CardShock Study Group

**Affiliations:** 1 Unit of Cardiovascular Research, Minerva Foundation Institute for Medical Research, 00029 Helsinki, Finland; heli.segersvard@helsinki.fi (H.S.); ilkka.tikkanen@helsinki.fi (I.T.); paivi.lakkisto@helsinki.fi (P.L.); 2 Department of Cardiology, University of Helsinki and Heart and Lung Center, Helsinki University Hospital, 00029 Helsinki, Finland; toni.jantti@hus.fi (T.J.); heli.tolppanen@gmail.com (H.T.); tuukka.tarvasmaki@hus.fi (T.T.); johan.lassus@hus.fi (J.L.); 3 Cardiovascular Research Unit, Department of Population Health, Luxembourg Institute of Health, L-1445 Strassen, Luxembourg; melanie.vausort@lih.lu (M.V.); yvan.devaux@lih.lu (Y.D.); 4 Intensive Cardiac Care Unit, Cardiology Department, Biomedical Research Institute IIB-SantPau, Hospital de la Santa Creu i Sant Pau, Universidad Autónoma de Barcelona, 08041 Barcelona, Spain; asionis@santpau.cat; 5 Centro de Investigación Biomédica en Red de Enfermedades Cardiovasculares (CIBERCV), 28029 Madrid, Spain; 6 Abdominal Center, Nephrology, University of Helsinki and Helsinki University Hospital, 00029 Helsinki, Finland; 7 Division of Emergency Medicine, University of Helsinki and Department of Emergency Services and Medicine, Helsinki University Hospital, 00029 Helsinki, Finland; veli-pekka.harjola@hus.fi; 8 Department of Clinical Chemistry and Hematology, University of Helsinki and Helsinki University Hospital, 00029 Helsinki, Finland

**Keywords:** cardiogenic shock, microRNA, mortality, prognosis

## Abstract

Cardiogenic shock (CS) is a life-threatening emergency. New biomarkers are needed in order to detect patients at greater risk of adverse outcome. Our aim was to assess the characteristics of miR-21-5p, miR-122-5p, and miR-320a-3p in CS and evaluate the value of their expression levels in risk prediction. Circulating levels of miR-21-5p, miR-122-5p, and miR-320a-3p were measured from serial plasma samples of 179 patients during the first 5–10 days after detection of CS, derived from the CardShock study. Acute coronary syndrome was the most common cause (80%) of CS. Baseline (0 h) levels of miR-21-5p, miR-122-5p, and miR-320a-3p were all significantly elevated in nonsurvivors compared to survivors (*p* < 0.05 for all). Above median levels at 0h of each miRNA were each significantly associated with higher lactate and alanine aminotransferase levels and decreased glomerular filtration rates. After adjusting the multivariate regression analysis with established CS risk factors, miR-21-5p and miR-320a-3p levels above median at 0 h were independently associated with 90-day all-cause mortality (adjusted hazard ratio 1.8 (95% confidence interval 1.1–3.0), *p* = 0.018; adjusted hazard ratio 1.9 (95% confidence interval 1.2–3.2), *p* = 0.009, respectively). In conclusion, circulating plasma levels of miR-21-5p, miR-122-5p, and miR-320a-3p at baseline were all elevated in nonsurvivors of CS and associated with markers of hypoperfusion. Above median levels of miR-21-5p and miR-320a-3p at baseline appear to independently predict 90-day all-cause mortality. This indicates the potential of miRNAs as biomarkers for risk assessment in cardiogenic shock.

## 1. Introduction

Cardiogenic shock (CS) is an acute state of inadequate cardiac output, resulting in end-organ hypoperfusion, multisystem organ failure, and high in-hospital mortality [1,2,3,4]. The etiology of CS is most often acute coronary syndrome (ACS) (i.e., shock caused by acute myocardial infarction (AMI)), but several other cardiac emergencies (e.g., end-stage heart failure, acute severe mitral regurgitation, cardiac tamponade or rupture, isolated right ventricular failure, or prior severe valvular disease) can also cause CS [5,6].

MicroRNAs (miRNAs) are a type of small (≈22 nucleotides) noncoding RNA that regulate post-transcriptional gene expression through the RNA interference (RNAi) pathway [7]. In recent years, miRNAs have been found to be present in human plasma and serum. They have been shown to have remarkable resilience against degradation caused by endogenous plasma ribonuclease (RNase) activity or multiple freeze–thaw cycles [8]. The presence of stable extracellular miRNAs has sparked theorization of their possible role as potential novel biomarkers in various diseases, with promising findings already described in different cardiovascular diseases (CVD) and cancer types [9,10,11].

With regard to specific miRNAs that could be involved in the pathophysiology of CS, we considered miR-21-5p, miR-122-5p, and miR-320a-3p as possible candidates. miR-21-5p is expressed broadly in different human tissues and changes in its expression have been shown in various diseases, including CVD [12,13]. There is increasing evidence for miR-21-5p having a cardioprotective role in the heart after ischemia-reperfusion injury and increased expression levels of miR-21-5p in serum have been reported in elderly patients after AMI [14,15,16]. miR-122-5p has been found to be a largely liver-specific miRNA that is involved in hepatocyte homeostasis and lipid metabolism [17,18]. Circulating plasma levels of miR-122-5p have been shown to be elevated during hepatic damage and also in patients with acute heart failure (AHF) [19,20]. It is also one of the few miRNAs that has prior evidence of potential involvement in CS, as its plasma levels were found to be increased in a porcine model of CS [21]. MiR-320a-3p is not known to be a tissue-specific miRNA, but has been demonstrated to have an important role in various pathological cellular processes, one of which is regulation of cardiomyocyte apoptosis after I/R injury [22,23]. Additionally, circulating plasma levels of miR-320a-3p were shown to be elevated in patients after AMI [24].

Currently, the number of studies regarding miRNAs in cardiogenic shock is fairly limited. Therefore, the aim of our study was to investigate the patient profiles associated with elevated levels of circulating miR-21-5p, miR-122-5p, and miR-320a-3p as well as their prognostic potential, in order to broaden our understanding of miRNAs in CS.

## 2. Results

### 2.1. miRNA Association with Baseline Characteristics

Blood samples from a total of 179 patients were used in the study cohort. The mean age was 66 years and 26% were women. The 90-day all-cause mortality was 42%. Acute coronary syndrome (ACS) was the most common cause of CS (80%), with severe chronic heart failure (12%) and valvular causes (7%) making up the majority of non-ACS causes of CS. Patients whose expression level of at least one of the three investigated miRNAs (miR-21-5p, miR-122-5p, or miR-320a-3p) was above median had a lower estimated glomerular filtration rate (eGFR) and also higher levels of lactate and alanine aminotransferase (ALT) (Table 1). The levels of miR-21-5p, miR-122-5p, and miR-320a-3p at baseline were each significantly higher in nonsurvivors compared with survivors (0.136 arbitrary units (AU) (interquartile range (IQR) 0.059–0.343) vs. 0.067 AU (IQR 0.030–0.200), *p* = 0.006; 0.086 AU (IQR 0.013–0.728) vs. 0.022 AU (IQR 0.002–0.191), *p* = 0.007; 0.035 AU (IQR 0.017–0.080) vs. 0.019 AU (IQR 0.007–0.066), *p* = 0.017; respectively). Additionally, the baseline miR-320a-3p levels were significantly higher in CS patients with ACS etiology compared to patients with non-ACS etiology (0.025 AU (IQR 0.011–0.091) vs. 0.017 AU (IQR 0.006–0.025), *p* = 0.017).

### 2.2. miRNA Characteristics, Mortality, and Prognostic Value at Baseline

Differences in biochemical and clinical findings between levels of individual miRNAs were also observed (Table 2). Patients with miR-21-5p levels above median at baseline developed more often acute kidney injury compared with patients with miR-21-5p levels below median. Patients with miR-122-5p levels above median at baseline presented more frequently with an altered mental state and had higher ALT and total bilirubin levels than patients with miR-122-5p levels below median. In addition, patients with miR-320a-3p levels above median at baseline had lower cardiac index compared with patients with miR-320a-3p levels below median.

A very high positive correlation was observed between miR-21-5p and miR-320a-3p levels at baseline as well as a high positive correlation between miR-122-5p levels and both miR-21-5p and miR-320a-3p levels at baseline (Table 3). There was also a high positive correlation between baseline miR-122-5p level and ALT, a moderate positive correlation between miR-122-5p level and lactate, and a low positive correlation between miR-21-5p and both ALT and lactate, as well as miR-320a-3p and both ALT and lactate.

**Figure 1:**
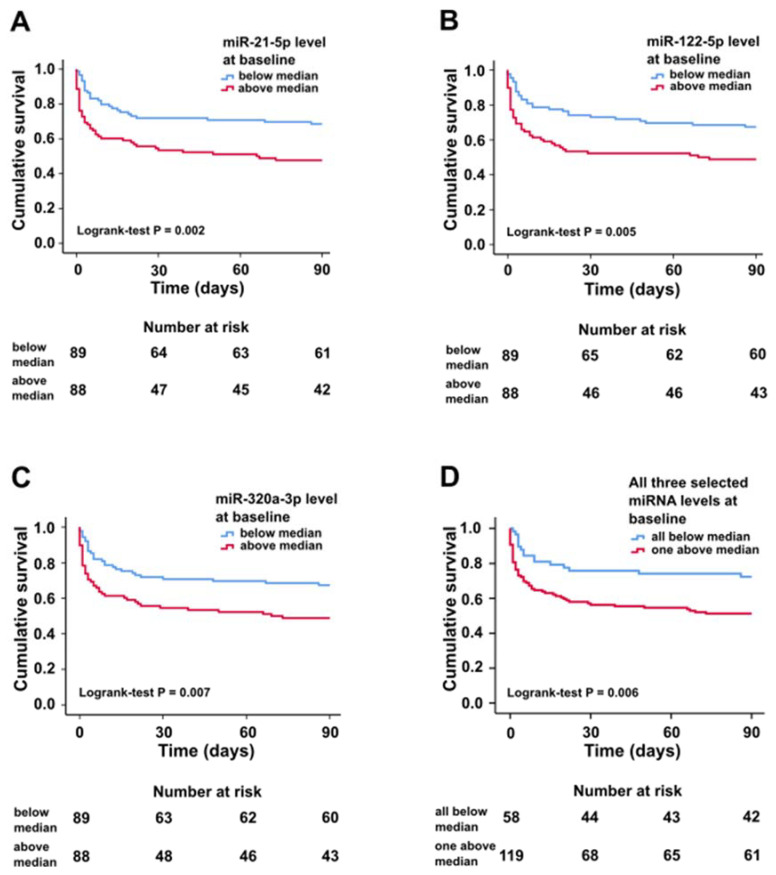
Kaplan–Meier survival curves for patients with plasma miRNA expression levels above and below median at baseline for miR-21-5p (**A**), miR-122-5p (**B**), miR-320a-3p (**C**), and all three selected miRNAs either below median or at least one above median at baseline (**D**).

In Kaplan–Meier survival analysis, circulating miR-21-5p, miR-122-5p, and miR-320a-3p levels above median were associated with higher 90-day all-cause mortality (Figure 1).

In Cox regression analysis, miR-21-5p, miR-122-5p, and miR-320a-3p levels above median were each associated with 90-day all-cause mortality with an unadjusted hazard ratio (HR) of 2.0 (95% CI 1.3–3.2, *p* = 0.003), 1.9 (95% CI 1.2–3.0, *p* = 0.007), and 1.9 (95% CI 1.2–3.0, *p* = 0.009), respectively. After adjusting the model with the CardShock risk score variables and ALT at baseline, we found miR-21-5p and miR-320a-3p levels above median to be both independently associated with 90-day all-cause mortality, while miR-122-5p levels above median were not (Table 4). Addition of either miR-21-5p or miR-320a-3p level above baseline as a variable to the CardShock risk score improved its predictive power of 90-day all-cause mortality as shown by comparison of nested Cox models (χ^2^ = 5.4, *p* = 0.020 and χ^2^ = 6.0, *p* = 0.014, respectively; c-index for model without miR-21-5p/miR-320a-3p = 0.805, c-index for model with miR-21-5p = 0.814 and c-index for model with miR-320a-3p = 0.817).

### 2.3. miRNA Levels at Later Time Points

**Figure 2:**
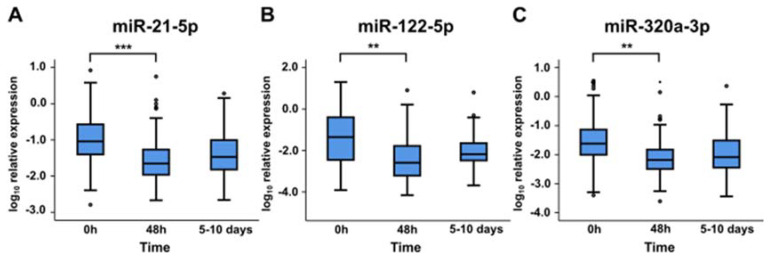
Relative expression levels of miR-21-5p (**A**), miR-122-5p (**B**), and miR-320a-3p (**C**) in cardiogenic shock patients at 0 h, 48 h, and 5–10 days. **, *p*-value < 0.01; ***, *p*-value < 0.001.

Expression levels of miR-21-5p, miR-122-5p, and miR-320a-3p above median at 48 h or 5–10 days were not associated with 90-day all-cause mortality. All miRNAs showed significant decreases in their expression levels in plasma between baseline and 48 h. Expression levels seemed to increase slightly between 48 h and 5–10 days, although this change was not statistically significant (Figure 2).

In the study cohort, both miR-21-5p and miR-320a-3p levels above median at baseline were associated with higher high-sensitivity troponin T (hsTnT) values at 12h in patients with ACS etiology (9514 vs. 3656 ng/L, *p* = 0.006, 8055 vs. 3556 ng/L, *p* = 0.009, respectively), while miR-122-5p levels were not. Furthermore, miR-320a-3p levels at 48h were associated with higher hsTnT values at 48h in patients with ACS etiology (5264 vs. 3158 ng/L, *p* = 0.049). We were unable to show similar associations between miRNA levels and hsTnT values in patients with non-ACS etiology. Additionally, expression levels of miR-320a-3p at 5–10 days were significantly higher in nonsurvivors compared with survivors (median 0.012 AU (IQR 0.007–0.063) vs. 0.007 (IQR 0.003–0.023), *p* = 0.034).

## 3. Discussion

In the present study, we show that circulating levels of miR-21-5p, miR-122-5p, and miR-320a-3p are elevated in nonsurvivors of CS compared to survivors. Above median levels of miR-21-5p, miR-122-5p and miR-320a-3p were associated with known markers of hypoperfusion. Additionally, we showed that both miR-21-5p and miR-320a-3p were each independently associated with 90-day all-cause mortality in CS after adjustment with established CS risk factors. Both miRNAs also improved the predictive power regarding 90-day all-cause mortality when either one was added as a variable to the CardShock risk score model. To our knowledge, this study is one of the first to investigate the characteristics and association with mortality of these miRNAs in CS and the first one to include patients with non-ACS etiology of CS.

There are only a few published studies that have investigated the role of miRNAs in CS. In addition, in most of these studies CS patients were only a small subgroup of study cohorts comprised of mainly ACS patients, which limits their applicability regarding CS [25,26,27]. We have previously found that above median circulating levels of miR-423-5p at baseline were independently associated with 90-day all-cause mortality in CS [28]. In addition, one previous study has evaluated the dynamics of miR-21-5p, miR-122-5p, and miR-320a-3p in CS [29]. In contrast with the present study, they found no association between serum miRNA levels and all-cause mortality. However, it is worth noting that the blood samples were processed differently from ours (serum vs. plasma samples), their study cohort was significantly smaller compared to ours (43 vs. 179 patients) and it consisted of only CS patients with ACS etiology.

MiR-21-5p is expressed broadly in various human tissues and is one of the most widely studied miRNA. While it is not a heart-specific miRNA, multiple studies have linked miR-21-5p to various CVDs and it seems to have a cardioprotective role in the initial stages of I/R injury [12,30]. A few in vitro studies have hypothesized this cardioprotective role during ischemia to be mediated by upregulating HIF-1α through the regulation of the PTEN/Akt pathway as well as inhibiting its proapoptotic target gene PDCD4 [31,32]. In our study, we found baseline miR-21-5p levels to be associated with 12h hsTnT values both overall and in patients with ACS etiology of CS. Expression levels of miR-21-5p also decreased significantly from baseline to 48 h. These findings could indicate a role for miR-21-5p in regulating cardiac injury and its release from the injured heart. However, our study also found a weak association of miR-21-5p levels with ALT, lactate, and eGFR at baseline. As miR-21-5p is a ubiquitously expressed miRNA, systemic hypoperfusion induced by CS can cause cellular injury and subsequent release of miR-21-5p from other organs as well. Nevertheless, considering the findings of previous studies and independent associated of miR-21-5p with 90-day all-cause mortality in our study, miR-21-5p shows potential as a prognostic biomarker in CS.

Not much is yet known of the role of miR-320a-3p in cardiovascular diseases, as most of the previous studies investigating this miRNA have focused on its role in various cancer types [33,34,35]. However, there is an increasing number of studies linking it to regulation of cardiomyocyte apoptosis during I/R injury. Upregulation of miR-320a-3p in cardiomyocytes appears to have a proapoptotic effect, with downregulation of Hsp20 and AKIP1 proposed as possible cellular level mechanisms [22,23]. It is also one of the few miRNAs that was reported to be elevated in plasma after AMI [24]. In our study, patients with ACS etiology of CS had higher baseline miR-320a-3p levels compared to non-ACS patients and baseline miR-320a-3p levels were associated with hsTnT values at 12h both overall and in ACS patients. Furthermore, patients with miR-320a-3p levels above median at baseline had lower cardiac index when compared to patients with below median miR-320a-3p levels. These findings could indicate a potential role of miR-320a-3p in the pathogenesis of cardiac injury. Results from other studies seem to support this hypothesis, as a recent clinical study found circulating miR-320a-3p to be positively associated with left ventricular adverse remodeling after AMI [36]. Additionally, the only previous study investigating miR-320a-3p in CS showed that expression levels peaked at 12h, which fits with our findings of baseline miR-320a-3p associating with hsTnT levels at 12h [29]. It should be mentioned, that miR-320a-3p is not a heart-specific miRNA and as with miR-21-5p, the systemic hypoperfusion may cause release of miR-320a-3p from a variety of different cell types. However, miR-320a-3p appears to be the most cardiac-associated miRNA of the three studied here. Given that it also independently associated with 90-day all-cause mortality, additional studies investigating the role of miR-320a-3p as a potential prognostic cardiac biomarker are merited going forward.

MiR-122-5p is one of the few miRNAs that has been previously studied in CS and although these studies showed miR-122-5p to be elevated during CS, they were unable to show association between miR-122-5p and mortality [21,25,27]. In our study we found that miR-122-5p levels at baseline were elevated in nonsurvivors of CS, but like the studies before, we were unable to show independent association between circulating miR-122-5p levels and mortality in CS. In our study, miR-122-5p was associated with multiple signs and biomarkers of hypoperfusion. The elevated baseline miR-122-5p levels in nonsurvivors of CS are most likely due to hepatocyte injury caused by low cardiac output and subsequent liver congestion as previously hypothesized [20]. This theory is further supported by our findings, as miR-122-5p showed strong correlation between ALT and no significant difference in its plasma levels could be found between ACS and non-ACS patients. MiR-122-5p had the strongest correlation between lactate in our study and this is supported by a previous study, where miR-122-5p was independently associated with lactate [27]. Our study also showed that altered mental state, a common sign of severe systemic hypoperfusion [37], was more common in patients with elevated miR-122-5p levels at baseline. Considering these findings, it is possible that miR-122-5p could be used as a general organ hypoperfusion marker regardless of the etiology of shock.

As mentioned previously, both miR-21-5p and miR-320a-3p appear to improve the predictive power of the CardShock risk score model. Although these improvements were fairly modest, they highlight the potential of miRNAs as prognostic biomarkers in CS. Several limitations and unanswered questions still remain before miRNAs can be used as diagnostic biomarkers. The lack of normalization methods and analytical standards for circulating miRNAs limits potential interstudy comparability [38]. Little is known of how the levels of circulating miRNAs are regulated. For instance, renal clearance may have a role in miRNA excretion, but previous studies investigating this have had conflicting results [39,40]. Despite these challenges, there are several encouraging studies where the expression levels of multiple miRNAs are used to form disease-specific panels resulting in improved diagnostic efficiency [41]. Indeed, our findings show promise that a miRNA panel specific for CS could also be created and used in the future for better prognosis assessment of CS patients.

As a limitation, our study could have benefitted from additional time points, as there is some evidence of altering functions of miRNAs in tissues depending on the time point, as previously mentioned [42]. The proportion of patients whose cardiac index was measured was fairly small in this study. Pulmonary artery catheter was used at the discretion of the treating physician, and as an invasive procedure with no proven treatment benefit was used only in a selected group of patients.

In conclusion, above median levels of miR-21-5p, miR-122-5p and miR-320a-3p were associated with higher mortality in CS, as well as general markers of hypoperfusion. Both miR-21-5p and miR-320a-3p levels above median at baseline were independently associated with 90-day all-cause mortality. Our findings highlight the potential role of miRNAs as additional prognostic biomarkers in CS, which could help develop more personalized management of this complex condition. In the future, additional studies investigating their roles in the pathophysiology of CS and other CVDs are warranted.

## 4. Materials and Methods

This study was a predefined substudy of the CardShock study. The CardShock study (ClinicalTrials.gov identifier: NCT01374867) was a multicenter, observational, and prospective study of CS, conducted in nine tertiary hospitals from eight European countries between October 2010 and December 2012. The study was approved by the following local ethics committees at the participating centers: Helsinki: The Ethics Committee, Department of Medicine, The Hospital District of Helsinki and Uusimaa (117/13/03/01/2010, 27 October 2010); Athens: Ethics Committee of Attikon University Hospital; Barcelona: Health Research Ethics Committee of the Hospital de Sant Pau; Brescia: Ethics Committee of the Province of Brescia; Brno: Ethic committee of University Hospital Brno; Porto: Ethics committee of S. João Hospital Center/Porto Medical School; Rome: Ethical Committee Sant’Andrea Hospital; Warsaw: Local Bioethics Committee of the Institute of Cardiology. Copenhagen: The study was approved by the Danish Protection Agency with reference number GEH-2014-013; I-Suite number: 02731. The study was conducted in accordance with the Declaration of Helsinki. Written informed consent was obtained from the patient or next of kin if the patient was unable to give the consent on admission.

Study enrolment required patients to be over 18 years old and within 6 h of CS identification. Definition of CS and study inclusion criteria were as follows: (1) acute cardiac cause, (2) systolic blood pressure <90 mmHg, and (3) one or more signs of organ hypoperfusion (altered mental state, blood lactate >2 mmol/L, cold extremities or oliguria <0.5 mL/kg/h for the previous 6 h). Exclusion criteria were shock either caused by hemodynamically significant arrythmias or presenting after cardiac surgery. For further details regarding the study population and main findings of the CardShock study, see Harjola et al. [6].

Plasma samples were collected at baseline (0 h), 48 h, and at discharge from the intensive care unit (ICU) or cardiac care unit (CCU) (i.e., 5–10 days after identification of CS) in EDTA tubes. Plasma was immediately separated, frozen in aliquots, and stored at −80 °C. The number of available samples was 179 at baseline (four patients had missing samples), 126 at 48 h (28 patients died before this timepoint and 29 patients had missing samples), and 75 at discharge from ICU/CCU (42 patients died before this timepoint and 66 patients had missing samples). Echocardiography was performed per protocol and clinical characteristics were evaluated upon study enrolment. Alanine aminotransferase (ALT), C-reactive protein (CRP), creatinine, high-sensitivity troponin T (hsTnT), N-terminal fragment of pro-B-type natriuretic peptide (NT-proBNP), and total bilirubin (Roche Diagnostics, Basel, Switzerland) were analyzed centrally at an accredited laboratory (ISLAB, Kuopio, Finland). Arterial blood lactate was analyzed locally. Out of the 179 patients that we studied, 37 patients had pulmonary artery catheters at baseline and their cardiac indexes were calculated using the thermodilution technique. The eGFR of patients were calculated using the Chronic Kidney Disease Epidemiology Collaboration (CKD-EPI) equation [43]. Acute kidney injury (AKI) was defined using the KDIGO creatinine-based criteria, as described previously [44]. The primary endpoint for this study was 90-day all-cause mortality.

Total RNA extraction and miRNA expression levels were assessed using methods and materials as previously described [45]. Briefly, total RNA was extracted from plasma samples using the mirVana PARIS kit (Ambion, Applied Biosystem, Lennik, Belgium). Spike-in synthetic *Caenorhabditis elegans* miRNA (cel-miR-39) (Qiagen, Venlo, The Netherlands) was added as normalization control. DNase treatment was used to remove potential genomic DNA contamination, after which reverse transcription was performed with the miScript PCR System (Qiagen). Expression values yielded from quantitative real-time polymerase chain reaction (qPCR) were normalized using the threshold cycle (Ct) of the cel-miR-39 control and calculated using the formula: 2^(Ct cel-miR-39-Ct miR of interest)^. The Ct values of all studied miRNAs were below 30. A pool of all patient samples was used as an internal calibrator between all PCR plates for each miRNA.

Data are presented as numbers (n) and percentages (%) for categorical variables, as mean and standard deviation (SD) for normally distributed variables, or as median and interquartile range (IQR) for variables with a skewed distribution. Patients were dichotomized based on the median baseline miRNA expression levels, both as an aggregate of the selected three miRNAs and also each miRNA separately. Between group comparisons were performed with chi-square test for categorical variables and Student’s *t*-test or Mann–Whitney U-test for continuous variables, as appropriate. Paired sample *t*-test was used to compare groups between different timepoints. Association between continuous variables was assessed using Spearman’s rank-order correlation. Differences in survival between groups were determined using Kaplan–Meier survival plots and the log-rank test. Univariable and multivariable Cox proportional hazards models were used to assess association between variables and 90-day all-cause mortality. The assumption of proportional hazards was verified graphically by evaluating the parallelism of each variable’s log-log survival curves. Multivariable analysis adjustments were made with the following variables: the CardShock risk score variables [6] and ALT at baseline. Comparison of nested Cox regression models was performed using the likelihood ratio chi-square test. The results from the regression models are presented as hazard ratios (HR) with 95% confidence intervals (CI). The evaluation of the discriminative ability of the risk prediction models was performed by comparing the respective areas under the receiver operating characteristic (ROC) curve (AUC) i.e., their concordance index (c-index). We considered two-sided *p*-value of < 0.05 to be statistically significant. All statistical analyses of data were performed using SPSS statistical software version 25 (IBM Corp., Armonk, NY, USA).

## Figures and Tables

**Table 1:** Patient characteristics, clinical and biochemical findings in relation to miR-21-5p, miR-122-5p, and miR-320a-3p at baseline.

Variable	All (n = 179)	All Selected miRNA Below Median (n = 59)	One or More of Selected miRNA above Median (n = 120)	*p*-Value
Age, years	66 (12)	64 (13)	67 (12)	0.096
Women, n (%)	47 (26)	11 (19)	36 (30)	0.105
BMI, kg/m^2^	26.9 (4.2)	26.2 (4.3)	27.3 (4.1)	0.101
Prior MI, n (%)	45 (25)	14 (24)	31 (26)	0.757
Prior CABG, n (%)	11 (6)	5 (9)	6 (5)	0.363
**Clinical Characteristics**				
ACS etiology, n (%)	143 (80)	45 (76)	98 (82)	0.395
Altered mental state at presentation, n (%)	118 (67)	34 (58)	84 (71)	0.719
Oliguria, n (%)	94 (53)	29 (49)	65 (56)	0.424
LVEF, %	33 (14)	33 (14)	33 (14)	0.734
**Biochemical Findings**				
eGFR, mL/min/1.73 m^2^	63 (30)	72 (29)	58 (29)	0.003
hsTnT, ng/L	2190 (388–5418)	1473 (407–5419)	2427 (386–5417)	0.645
NT-proBNP, ng/L	2710 (585–9434)	2475 (942–7487)	2759 (563–9716)	0.888
ALT, U/L	44 (20–92)	21 (11–42)	66 (29–129)	<0.001
Blood lactate, mmol/L	2.7 (1.7–5.7)	2.1 (1.4–3.1)	3.4 (2.1–6.7)	<0.001
CRP, mg/L	16 (4–54)	17 (5–48)	15 (4–60)	0.925

Results are presented as numbers (n) and percentages (%), mean ± SD for normally distributed variables and median with IQR for non-normally distributed variables. BMI, body mass index; MI, myocardial infarction; CABG, coronary artery bypass grafting; ACS, acute coronary syndrome; LVEF, left ventricular ejection fraction; eGFR, estimated glomerular filtration rate; hsTnT, high-sensitivity troponin T; NT-proBNP, N-terminal fragment of pro-B-type natriuretic peptide; ALT, alanine aminotransferase; CRP, C-reactive protein.

**Table 2:** Patient characteristics, clinical and biochemical findings in relation to miR-21-5p, miR-122-5p, and miR-320a-3p at baseline.

Variable	All (n = 179)	miRNA Below Median (n = 59)	miRNA above Median (n = 120)	*p*-Value
**miR-21-5p**				
eGFR, mL/min/1.73 m^2^	63 (30)	70 (31)	55 (26)	0.001
Blood lactate, mmol/L	2.7 (1.7–5.7)	2.2 (1.3–3.2)	3.7 (2.3–6.7)	0.001
ALT, U/L	44 (20–92)	31 (17–66)	71 (28–129)	<0.001
In hospital mortality, n (%)	67 (37)	27 (30)	40 (45)	0.038
Acute kidney injury, * n (%)	67 (44)	29 (35)	38 (55)	0.015
90-day mortality, n (%)	74 (42)	28 (31)	46 (52)	0.005
**miR-122-5p**				
Altered mental state, n (%)	118 (67)	50 (56)	68 (77)	0.003
eGFR, mL/min/1.73 m^2^	63 (30)	67 (29)	58 (30)	0.041
Blood lactate, mmol/L	2.7 (1.7–5.7)	2.1 (1.4–3.1)	5.0 (2.4–8.2)	<0.001
ALT, U/L	44 (20–92)	21 (12–42)	88 (49–175)	<0.001
Total bilirubin, μμmol/L	9.6 (5.7–15.4)	8.6 (5.7–12.7)	10.5 (6.1–20.3)	0.047
In hospital mortality; n (%)	67 (37)	26 (29)	41 (46)	0.018
90-day mortality; n (%)	74 (42)	29 (33)	45 (51)	0.012
**miR-320a-3p**				
Cardiac index, # L/min/m^2^	2.2 (0.9)	2.5 (1.0)	1.8 (0.6)	0.021
eGFR, mL/min/1.73 m^2^	63 (30)	68 (30)	58 (28)	0.024
Blood lactate, mmol/L	2.7 (1.7–5.7)	2.4 (1.5–4.3)	3.5 (2.1–6.5)	0.015
ALT, U/L	44 (20–92)	31 (17–69)	58 (29–129)	<0.001
90-day mortality; n (%)	74 (42)	29 (33)	45 (51)	0.012

Results are presented as numbers (n) and percentages (%), mean ± SD for normally distributed variables and median with IQR for non-normally distributed variables. ALT, alanine aminotransferase; eGFR, estimated glomerular filtration rate. *, n = 151; #, n = 37.

**Table 3:** Spearman correlation coefficients of miRNAs and biochemical values at baseline.

	miR-21-5p	miR-122-5p	miR-320a-3p	ALT	Creatinine	Lactate	hsTnT	NT-proBNP
miR-21-5p	1.00	0.66 ***	0.90 ***	0.39 ***	0.17 *	0.28 ***	0.12	−0.02
miR-122-5p		1.00	0.67 ***	0.72 ***	0.16 *	0.50 ***	0.00	−0.08
miR-320a-3p			1.00	0.39 ***	0.18 *	0.25 **	0.11	−0.08

ALT, alanine aminotransferase; hsTNT, high-sensitivity troponin T; NT-proBNP, N-terminal fragment of pro-B-type natriuretic peptide. *, *p*-value < 0.05; **, *p*-value < 0.01; ***, *p*-value < 0.001.

**Table 4:** Multivariable Cox regression analysis showing hazard ratios of individual miRNA levels when adjusted with the CardShock risk score variables and ALT at baseline.

Variable	Hazard Ratio (95% CI)	*p*-Value
**miR-21-5p**		
miR-21-5p level above median	2.10 (1.26–3.49)	0.004
Age	1.02 (0.99–1.04)	0.183
Altered mental state	1.92 (1.01–3.65)	0.048
Previous MI or CABG	1.74 (1.05–2.87)	0.031
ACS etiology	1.52 (0.76–3.04)	0.240
LVEF	0.97 (0.95–0.99)	0.002
Lactate	1.08 (1.03–1.14)	0.001
eGFR	0.99 (0.98–1.00)	0.049
ALT	1.00 (1.00–1.00)	0.301
**miR-122-5p**		
miR-122-5p level above median	1.33 (0.76–2.34)	0.321
Age	1.02 (0.99–1.04)	0.250
Altered mental state	1.71 (0.88–3.29)	0.111
Previous MI or CABG	1.77 (1.06–2.94)	0.028
ACS etiology	1.59 (0.78–3.23)	0.198
LVEF	0.98 (0.96–1.00)	0.014
Lactate	1.09 (1.03–1.14)	0.001
eGFR	0.99 (0.98–1.00)	0.038
ALT	1.00 (1.00–1.00)	0.481
**miR-320a-3p**		
miR-320a-3p level above median	2.01 (1.21–3.31)	0.007
Age	1.02 (0.99–1.05)	0.140
Altered mental state	1.72 (0.91–3.27)	0.098
Previous MI or CABG	1.79 (1.08–2.97)	0.024
ACS etiology	1.48 (0.74–2.98)	0.271
LVEF	0.97 (0.95–0.99)	0.006
Lactate	1.09 (1.04–1.14)	0.001
eGFR	0.99 (0.98–1.00)	0.051
ALT	1.00 (1.00–1.00)	0.339

MI, myocardial infarction; CABG, coronary artery bypass grafting; ACS acute coronary syndrome; LVEF, left ventricular ejection fraction; eGFR, estimated glomerular filtration rate; ALT, alanine aminotransferase; CI, confidence interval.
